# Persistent *Plasmodium falciparum* and *Plasmodium vivax* infections in a western Cambodian population: implications for prevention, treatment and elimination strategies

**DOI:** 10.1186/s12936-016-1224-7

**Published:** 2016-03-24

**Authors:** Rupam Tripura, Thomas J. Peto, Jeremy Chalk, Sue J. Lee, Pasathorn Sirithiranont, Chea Nguon, Mehul Dhorda, Lorenz von Seidlein, Richard J. Maude, Nicholas P. J. Day, Mallika Imwong, Nicholas J. White, Arjen M. Dondorp

**Affiliations:** Mahidol Oxford Tropical Medicine Research Unit, Faculty of Tropical Medicine, Mahidol University, Bangkok, Thailand; Centre for Tropical Medicine and Global Health, Nuffield Department of Medicine, University of Oxford, Oxford, UK; National Centre for Parasitology, Entomology, and Malaria Control (CNM), Trapeng Svay Village, Sangkat Phnom Penh Thmei, Khan Sen Sok, Phnom Penh, Cambodia; WorldWide Antimalarial Resistance Network, Asia Regional Centre, Faculty of Tropical Medicine, Mahidol University, Bangkok, Thailand; Department of Epidemiology, Harvard T. H. Chan School of Public Health, Boston, MA 02115 USA; Department of Molecular Tropical Medicine and Genetics, Faculty of Tropical Medicine, Mahidol University, Bangkok, Thailand

**Keywords:** Malaria, Persistence, Cohort, Plasmodium, Falciparum, Vivax, Clearance, Artemisinins, Resistance, Pailin, Cambodia, PCR

## Abstract

**Background:**

Subclinical *Plasmodium* parasitaemia is an important reservoir for the transmission and persistence of malaria, particularly in low transmission areas.

**Methods:**

Using ultrasensitive quantitative PCR (uPCR) for the detection of parasitaemia, the entire population of three Cambodian villages in Pailin province were followed for 1 year at three-monthly intervals. A cohort of adult participants found initially to have asymptomatic malaria parasitaemia was followed monthly over the same period.

**Results:**

The initial cross sectional survey in June 2013 (M0) of 1447 asymptomatic residents found that 32 (2.2 %) had *Plasmodium falciparum*, 48 (3.3 %) had *P. vivax*, 4 (0.3 %) had mixed infections and in 142/1447 (9.8 %) malaria was detected but there was insufficient DNA to identify the species (*Plasmodium. species*). Polymorphisms in the ‘K13-propeller’ associated with reduced susceptibility to artemisinin derivatives (C580Y) were found in 17/32 (51 %) *P. falciparum* strains. Monthly follow-up without treatment of 24 adult participants with asymptomatic mono or mixed *P. falciparum* infections found that 3/24 (13 %) remained parasitaemic for 2–4 months, whereas the remaining 21/24 (87 %) participants had cleared their parasitaemia after 1 month. In contrast, 12/34 (35 %) adult participants with *P. vivax* mono-infection at M0 had malaria parasites (*P. vivax* or *P. sp.*) during four or more of the following 11 monthly surveys.

**Conclusions:**

This longitudinal survey in a low transmission setting shows limited duration of *P. falciparum* carriage, but prolonged carriage of *P. vivax* infections. Radical treatment of *P. vivax* infections by 8-aminoquinoline regimens may be required to eliminate all malaria from Cambodia.

*Trial registration* ClinicalTrials.gov NCT01872702

**Electronic supplementary material:**

The online version of this article (doi:10.1186/s12936-016-1224-7) contains supplementary material, which is available to authorized users.

## Background

Deforestation and standard malaria control efforts including early, appropriate case management and distribution of insecticide-treated bed nets have reduced malaria prevalence to historically low levels in much of Western Cambodia. Unfortunately these control measures have failed to contain the emergence of anti-malarial drug resistant strains of *Plasmodium falciparum* in an expanding geographical area [1, 2]. In areas with very low malaria transmission the large majority of malaria infections were thought to be symptomatic and hence accessible to passive detection and curative treatment [3]. Yet malaria has historically been difficult to eliminate even when most symptomatic patients received highly effective anti-malarial treatments. Symptomatic infections as a sole source of transmission cannot explain the virtual disappearance of malaria cases each year during the cool dry season and prompt return with the onset of rains. A significant sub-patent reservoir of *P. falciparum* carriers does explain both the epidemiology of malaria in these areas of seasonal malaria and why the current control and containment activities fail to contain resistant malaria [4, 5]. To understand and eliminate such a reservoir it is not only important to understand the prevalence at any one point in time but also the duration of individual infections.

Apparently healthy people who migrate to regions without malaria transmission and later give blood donations can remain infected asymptomatically with *P. falciparum* for up to 13 years [6]. Information on the distribution of persistent infections usually comes from cohort studies. Observational studies of untreated malaria patients were not uncommon during the last century. Lowe followed 16 people with untreated malaria in India 1934 [7]. Hill and co-workers followed 22 children with falciparum, malariae and/or vivax infections at weekly intervals during 1937 and 1938 in Aguas de Moura, Portugal who were only treated if symptomatic [8]. Earle et al. [9] followed 76 mostly untreated children in weekly intervals in Puerto Rico in 1939. McGregor and co-workers [10] studied falciparum infected, untreated children in The Gambia during the 1950s. Bruce-Chwatt [11] reported a cohort study of a group of West African adults in 1963. Bruce et al. [12] reported a study conducted in 1992 in which 70 people from a single village in Papua New Guinea (PNG) were sampled for up to 61 days. The advent of PCR based molecular diagnostics on low volume capillary blood spots allowed the documentation of persistent low-density *P. falciparum* infections in Sudan over more extended periods [8]. In 1997, a cohort of 43 recently malaria-infected Sudanese, aged from 9 to 53, agreed to donate fortnightly blood samples for the next 9 months. Of the 43 individuals, 16 (37 %) were found to maintain chronic *P. falciparum* infections for the follow-up period of 9 months. Together these studies demonstrated that individual patients can carry *P. falciparum* infections for weeks to years.

These historical field studies in endemic countries are not only limited by the detection threshold of light microscopy (the exception being the study in Sudan) but also by uncertainty regarding re-infections. The risk of reinfection is much reduced in very low or no transmission areas. A substantial contribution to the current understanding of the natural history of *Plasmodium* infections comes from the malaria therapy of neurosyphilis. In well-documented treatments patients with neurosyphilis were infected with *P. falciparum* or *P. vivax* in South Carolina and Georgia, USA during the period 1940–1963 [13, 14]. *Plasmodium falciparum* infections persisted (by microscopy) for a mean of 222 days the longest being 480 days [15]. Malaria naïve patients who received sporozoite-induced vivax infections and no anti-malarial therapy were found to have waves of parasitaemia during the follow-up period of 108 days [16]. In contrast to *P. falciparum* infections the dynamics of vivax infections are complicated by the liver reservoir of *P. vivax* hypnozoites, which cause periodic relapses and so contribute to the chronicity of parasitaemia [14].

The development of high volume ultrasensitive qPCR (uPCR) allows as few as 22 parasites/mL to be measured accurately compared to ~1000 parasites/mL using conventional low volume PCR methods [17]. Using uPCR for the detection of parasitaemia, the entire population of three Cambodian villages in Pailin province were followed over a year in order to describe the reservoir of sub-patent *Plasmodium* infection.

## Methods

### Study site and population

Pailin is an agricultural province adjacent to the Thailand border in western Cambodia. The villages are farming communities, which grow cash crops and fruit. Nearby forests are used as a source of plants and fruit, small game, bamboo and wood. Containment efforts in Cambodia have resulted in a marked decline in the incidence of clinical malaria over the last decade. Between 2004 and 2013 a 145-fold reduction in *P. falciparum* cases and a 4.8-fold reduction in *P. vivax* cases was observed [18]. Malaria control in Pailin has been based on case management by village health workers (VHW) or village malaria workers (VMW) and the distribution of long-lasting insecticide-treated bed nets (LLIN). There has been substantial replacement of forest by agriculture and rubber plantations, which could have contributed to the reduction in malaria transmission. Historically *P. falciparum* has been the dominant *Plasmodium* species causing malaria but more recently *P. vivax* infection has become predominant [18]. Malaria transmission is low and seasonal with entomological inoculation rates below one infectious bite/person/year [19, 20].

Western Cambodia has been the epicentre for the emergence of *P. falciparum* strains resistant against a range of anti-malarial drugs including chloroquine, sulfadoxine/pyrimethamine, artemisinins and piperaquine [21–26]. The first-line treatment for falciparum malaria through December 2013, the first 6 months of the study, was atovaquone/proguanil (Malarone^©^) which temporarily replaced artesunate–mefloquine, and was itself replaced in January 2014 with dihydroartemisinin/piperaquine (DHA/piperaquine) [27]. Vivax malaria is treated with a schizontocidal drug which is usually an artemisinin combination therapy (ACT). Radical cure for vivax malaria with primaquine after G6PD testing is recommended but as G6PD tests are unavailable this is seldom used. In the Pailin area the primary health care providers for febrile illness are the VMWs who are supervised by the local government health centre. The VMWs stock rapid diagnostic tests (RDTs) and ACT. Primary healthcare from VMWs is intended to be available 24 h a day and is free. Patients with a diagnosis other than malaria are referred to or go directly to a local health centre, which is approximately 6 km from the study villages and serves other villages in the surrounding area. Those who require hospitalization travel to the Pailin Referral Hospital, which is roughly 30 km from the study site. Malaria treatment by the private sector is prohibited in Pailin but pharmacies and drug sellers do stock anti-malarial drugs. In practice patients who believe that a malaria diagnosis is unlikely bypass the VMW.

In 2013, the Cambodian National Centre for Parasitology, Entomology and Malaria Control (CNM) and Mahidol-Oxford Tropical Medicine Research Unit (MORU) formed a research team based in Pailin Referral Hospital to investigate the prevalence of subclinical parasitaemia. Three study villages were selected for participation in the study on the basis of high relative incidence of clinical falciparum malaria in the village malaria worker records.

### Study procedures

In each village, a study committee was formed consisting of village leaders, VMWs, and volunteers. The committee assisted the study team in organizing the study and in engaging and mobilising the community. All households were geo-referenced by GPS and given a unique household number. In a census in April 2013 all residents were recorded, assigned a unique identification number and linked to a household number. In June 2013, the first of five cross-sectional surveys (M0) was conducted, followed by further surveys in October 2013 (M3), January 2014 (M6), April 2014 (M9) and June 2014 (M11). Before each survey, the population residing in the village at the time, including temporary residents and migrants were invited to participate. People moving into the villages between surveys for more than 2 weeks were included in the study.

Individual informed consent was obtained from adults and from parents or guardians of children aged less than 16 years. Information on demographics and household relationships were collected with a brief history of recent illness and travel. The tympanic temperature, weight, and height of all participants were measured. A venous blood sample (3 mL) from all individuals aged ≥5 years was collected in EDTA tubes, or 500 µL from children aged ≥6 months to 5 years. Participants with fever ≥37.5 °C were tested for malaria by rapid diagnostic test (Healgen malaria *P. falciparum*/Pan one-step RDT, Zhejiang Orient Biotech, China), and if positive were treated according to national guidelines [27].

Blood samples were stored on wet ice in the field and then transported within 9 h to a local laboratory in Pailin. Blood was centrifuged at 1500*g* for 10 min (Heraeus labfuge 400) to separate plasma and buffy coat from packed red blood cells (pRBC). 500 µL pRBC samples for uPCR analysis were then frozen and stored at −80 °C. Batches of frozen samples were transported monthly on dry ice to the Molecular Tropical Medicine laboratory in Bangkok, Thailand for uPCR analysis as described previously [17].

Participants aged 16 years and older who were found to be parasitaemic by uPCR were informed of their status and invited to join a study cohort. Cohort members were tested monthly for parasitaemia. The first survey (M1) was in August 2013. Subsequent cohort surveys were conducted in September 2013 (M2), November 2013 (M4), December 2013 (M5), February 2014 (M7), March 2014 (M8) and May 2014 (M10). After the study closed, the local health centre provided free treatment according to national guidelines to all participants with persistent parasitaemia.

### Laboratory methods

A detailed description, evaluation and validation of the high-volume uPCR methodology has been reported previously [17]. Briefly, the DNA template for detection and quantification of *Plasmodium* by PCR is purified from thawed pRBCs. The presence of malaria parasites and an estimate of the parasite numbers (genomes) in each sample are assessed by an absolute quantitative real-time PCR method using primers targeting the gene for 18S rRNA. A Quanti-Tect Multiplex PCR No ROX^®^ Kit (QIAGEN, Germany) was used for this purpose with the PCR reaction mixture and the cycling conditions as per manufacturer’s instructions. The probes used in the assay have been validated and are highly specific for *Plasmodium* species [28]. The lower limit of detection of this method is 22 parasites/mL of whole blood [29]. For samples containing parasite DNA by uPCR analysis, *Plasmodium* species detection was attempted using nested PCR protocols specific to *P. falciparum* (microsatellite marker Pk2), *P. vivax* (microsatellite marker 3.502) and *P. malariae* (18 s rRNA) as described previously [28, 30, 31]. Samples for which *Plasmodium* species could not be determined were reported as *Plasmodium species.*

To detect polymorphisms associated with reduced susceptibility to artemisinin derivatives the open-reading frame of the PF3D7_1343700 kelch propeller domain was amplified using a nested PCR protocol [1, 32]. Purified PCR products were sequenced at Macrogen, Republic of Korea and analysed using BioEdit version 7.1.3.0. using the 3D7 kelch13 sequence as reference (Accession: XM_001350122.1). The definition of single nucleotide polymorphisms (SNPs) was based on analytical approaches described previously [1, 33].

### Data management and statistical analyses

Data were collected on smartphones using ODK software [34] and then managed by importing into OpenClinica [35].

### Definitions

- At each quarterly survey, the numbers of residents and participants in the study villages changed as residents moved away, refused to participate, travelled, died, joined the village, were born or returned from travel. *Reasons for non-participation* were categorized as: moved away for more than 1 month, short travel (defined here as less than 1 month), refusal, unable, not known, or ineligible. Because the categories short travel, unable to attend and refusal can overlap, they were combined.
- The *ineligible* category included the severely ill, children less than 6 months of age or participants who did not consent to a blood draw.
- A *participant* was defined as an individual who participated in the survey and agreed to give a blood sample.
- *Coverage* was estimated as the percentage of residents who provided a blood sample for uPCR analysis (numerator) divided by the number of invited residents (denominator). The invited residents represent the de facto population in the village at the time of the respective survey.
- *Cohort* participants were those aged 16 years and older who were found to be parasitaemic at M0 and followed monthly during the study period.
- *Carriers* were a subgroup of the cohort defined for the purposes of this study as being parasitaemic during four or more surveys. The definition was derived from the frequency distribution. Carriers represent the top quartile of the frequency distribution, 75 % of the cohort had fewer than four episodes.

To determine independent predictors for parasitaemia within the adult cohort, a logistic regression model was developed wherein the order of observations for each subject was considered by month (M0–11). Each member of the cohort was fitted as a random effect to adjust for any dependence of repeated events. Risk factors investigated included, village, sex, occupation (farmer or not), and age (continuous). Time-varying risk factors (which were asked at each survey) included self-reported history of fever, self-reported history of malaria, travel (0/1), and bed net use (0/1). Potential interactions between covariates were also explored. To ensure good model fit, the quadrature approximation used in the random-effects estimators was checked. A *p* value <0.05 was considered statistically significant. All analyses were performed using Stata, version 13 (StataCorp, College Station, TX, USA).

### Additional data sources

Daily rainfall and maximum/minimum temperature data were collected from the Pailin Meteorological Office. Information on clinical (symptomatic) malaria episodes were collected by VMW, VHW and primary health centres for 2013–14.

### Ethics approval

The study was approved by the Cambodian National Ethics Committee for Health Research (0029 NECHR, dated 4th March 2013) and the Oxford Tropical Research Ethics Committee (1015-13, dated 29th April 2013).

## Results

### Study population

Details of 1758 residents were collected in the April 2013 census. Between June 2013 and June 2014 five surveys were conducted targeting all village residents. By the end of the surveillance period in June 2014 the number of residents who had been registered in the villages had increased to 2330. Over the same period many people had moved out of the villages, hence the number of people invited to the final M11 survey (1501) was similar to the number invited to the M0 survey (1540). Coverage in prevalence surveys ranged from 80–94 % of those present in the village at the time. The numbers of residents who were invited to participate, who participated or who did not participate are shown in Table 1.

**Table 1:** Assembly of study participants and coverage (see “Methods” section for definitions)

Survey	Cumulative enrolment/census	Moved away >1 month	Invited^a^	Travel^b^, unable refused	Reason not known	In-eligible	Participated^c^	Coverage^d^ (%)
M0	1758	218	1540	69	0	24	1447	94
M3	1992	469	1523	88	4	46	1385	91
M6	2125	573	1552	263	0	44	1245	80
M9	2230	845	1385	100	4	36	1245	90
M11	2330	829	1501	193	0	41	1266	84

^a^ Includes all villagers who were not away from the village for more than 1 month

^b^ Short travel away for <1 month

^c^ Provided a blood sample

^d^ *Coverage* participated/invited

### Participant characteristics

Data were collected on 2330 people residing during the whole or part of the study period in three participating villages. The median age was 22 years (range 3 months to 83 years). Overall, 52 % of residents were male, 39 % were children <16 years old and 15 % were ≤5 years old. There were 419 households with a median household size of five people. Most houses consisted of a large single room used for both daytime activities and for sleeping. More than 97 % of permanent villagers reported sleeping under a bed net at the time of each survey. The median time of residence was 6 years (range 1 month to 38 years). Demographic characteristics of the 2330 participants by uPCR result are shown in Table 2.

**Table 2:** Demographics of participants by uPCR result

	Positive^a^	%	Negative	%	No uPCR result	%	Total	%^b^	p value
N	333	14.3 %	1818	78.0 %	179	7.68 %	2330	100 %	
Sex									
Female	122	10.9 %	911	81.2 %	89	7.93 %	1122	48.2 %	
Male	211	17.5 %	907	75.1 %	90	7.45 %	1208	51.9 %	<0.0001
Age, years (median, range)	24	(7 months to 74 years)	21	(6 months to 80 years)	21	(3 months to 83 years)	22	(3 months to 83 years)	0.054
Village									
Krachap Leu	130	15.6 %	635	76.2 %	68	8.16 %	833	35.8 %	
O Kting	42	9.61 %	358	81.9 %	37	8.47 %	437	18.8 %	
Phnom Dambang	161	15.2 %	825	77.8 %	74	6.98 %	1060	45.5 %	0.029
Occupation									
Farms own land	142	19.2 %	539	72.7 %	60	8.10 %	741	31.8 %	
Hired farm labourer	66	10.8 %	512	83.9 %	32	5.25 %	610	26.2 %	
Child or student	116	13.1 %	700	79.1 %	69	7.80 %	885	38.0 %	
Other^c^	9	9.57 %	67	71.3 %	18	19.2 %	94	4.03 %	<0.0001

Numbers are frequency (%), unless otherwise specified

^a^ At least once during the study period

^b^ Column percentages

^c^ Military or police, own business, health worker, stays at home, retired or disabled, government staff, monk/nun, resort staff, NGO employee, teacher, village chief

### Seasonal distribution of parasitaemia

**Fig. 1:**
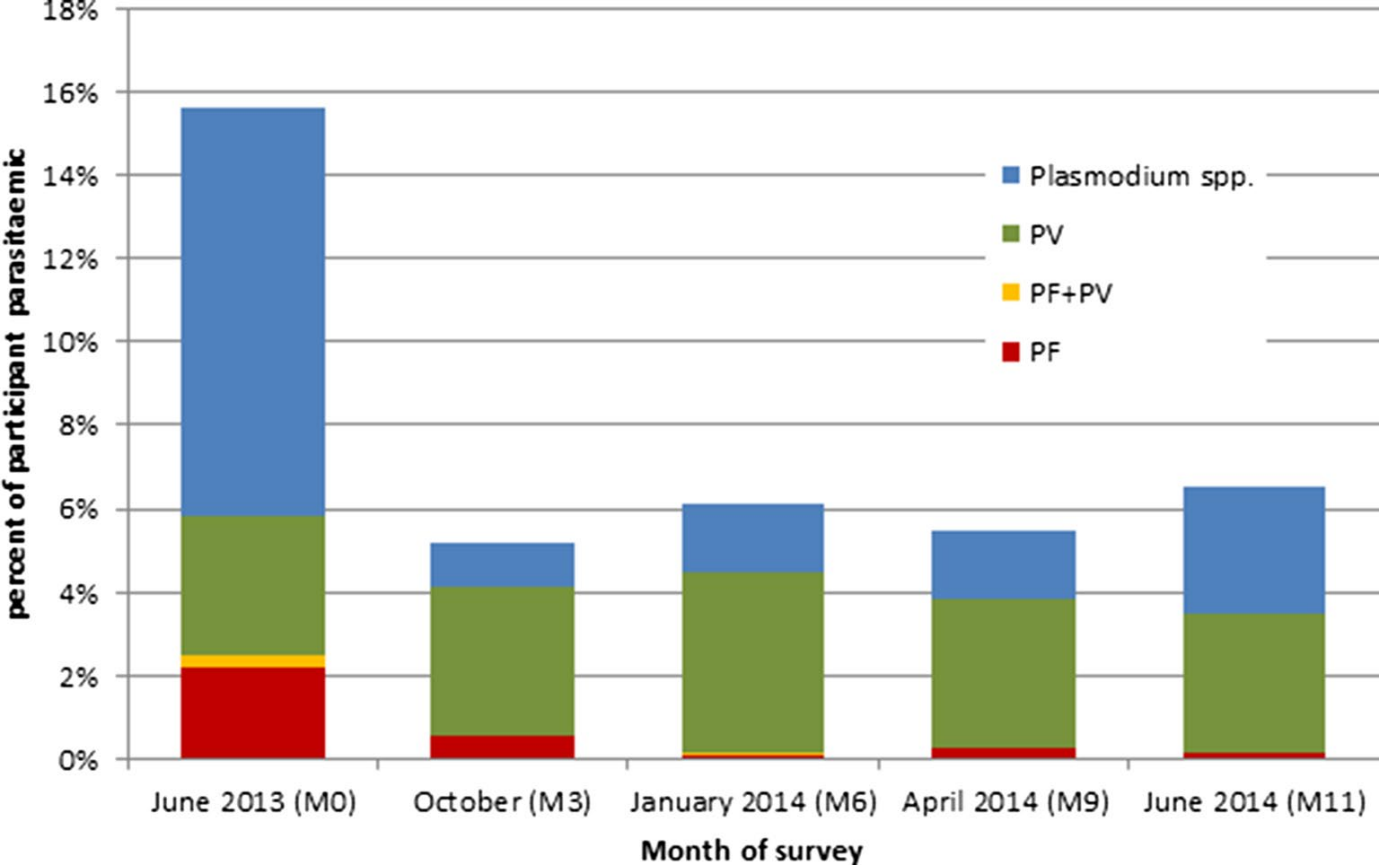
Parasite prevalence at 3-monthly surveys of the entire village

**Fig. 2:**
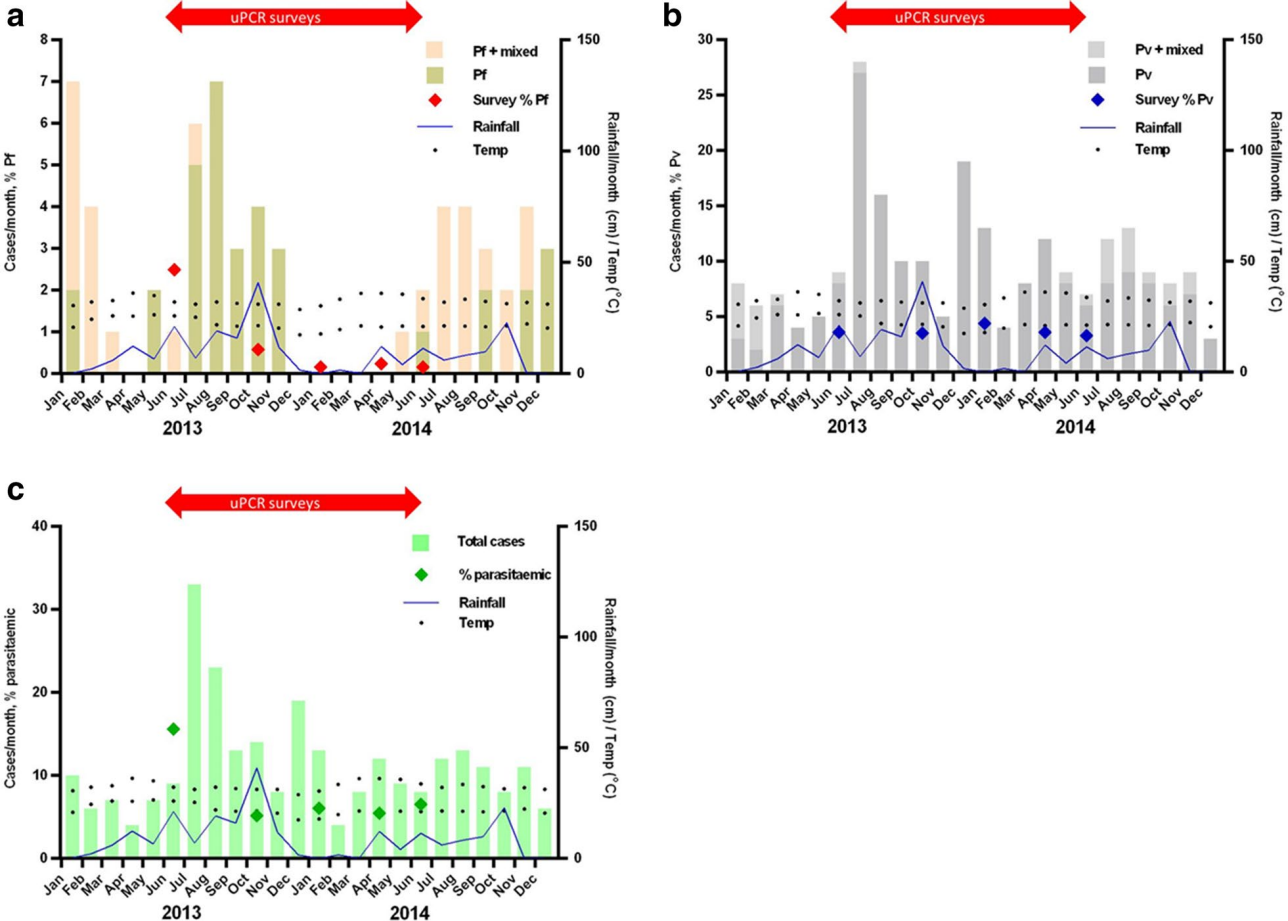
**a**, **b**, and **c** represent the prevalence of *Plasmodium falciparum* or mixed infections (**a**), *Plasmodium vivax* or mixed infections (**b**), and all species, including *Plasmodium species* which could not be determined (**c**). The *figures* represent a composite of uPCR data from cross-sectional surveys, clinical data collected by village malaria workers, and meteorological data collected by the Department of Meteorology, Ministry of Water Resources and Meteorology, Cambodia. The percentage of specimens found to be positive by uPCR is indicate by *diamonds*. The ambient min/max temperature range is indicated by *dots*. The daily rainfall in mm is shown as a *blue line*. uPCR data were collected during the study period June 2013 and June 2014 indicated by the *red arrow*. The clinical and meteorological data were collected between January 2013 and December 2014. Information on malaria episodes were collected by village malaria (VMW), mobile malaria workers (MMW) and primary health centres for 2013–14

The seasonal distribution of *P. falciparum,P. vivax* and all *Plasmodium* infections, including infections in which the species could not be identified (*P. species*) are shown in Fig. 1. Based on uPCR in June 2013 (M0) 32/1447 (2.2 %) residents had *P. falciparum* infections, 48 (3.3 %) had *P. vivax* infections, 4 (0.3 %) had mixed infections, and 142 (9.8 %) had malaria infection but the species could not be identified. No species other than *P. falciparum* or *P. vivax* were identified. The *P. falciparum* malaria prevalence dropped below 1 % by October 2013 (M3) and remained below 1 % during the following three surveys (M6, M9, M11). In contrast, *P. vivax* prevalence remained stable between 3 and 5 % during the study period. Overall the highest parasite prevalence (16 %; all species) was seen in June 2013 (early in the rainy season) and dropped to 5–7 % for the rest of the study period. Figure 2a–c illustrates, the relation between parasite prevalence estimated using uPCR, clinical cases diagnosed by VMW, VHW and primary health centres and meteorological variables. There was no detectable correlation between *Plasmodium* prevalence and rainfall or temperature.

### Resistance markers

The mutation C580Y in the PF3D7_1343700 kelch propeller domain which is associated with reduced susceptibility to artemisinin derivatives was found in 17/32 (51 %) *P. falciparum* strains. No other kelch polymorphisms were detected. The C580Y mutation was distributed heterogeneously between villages; it was found in 15/15 (100 %) samples from PDB village but in only 2/17 (12 %) samples from KL village.

### Cohort survey

**Fig. 3:**
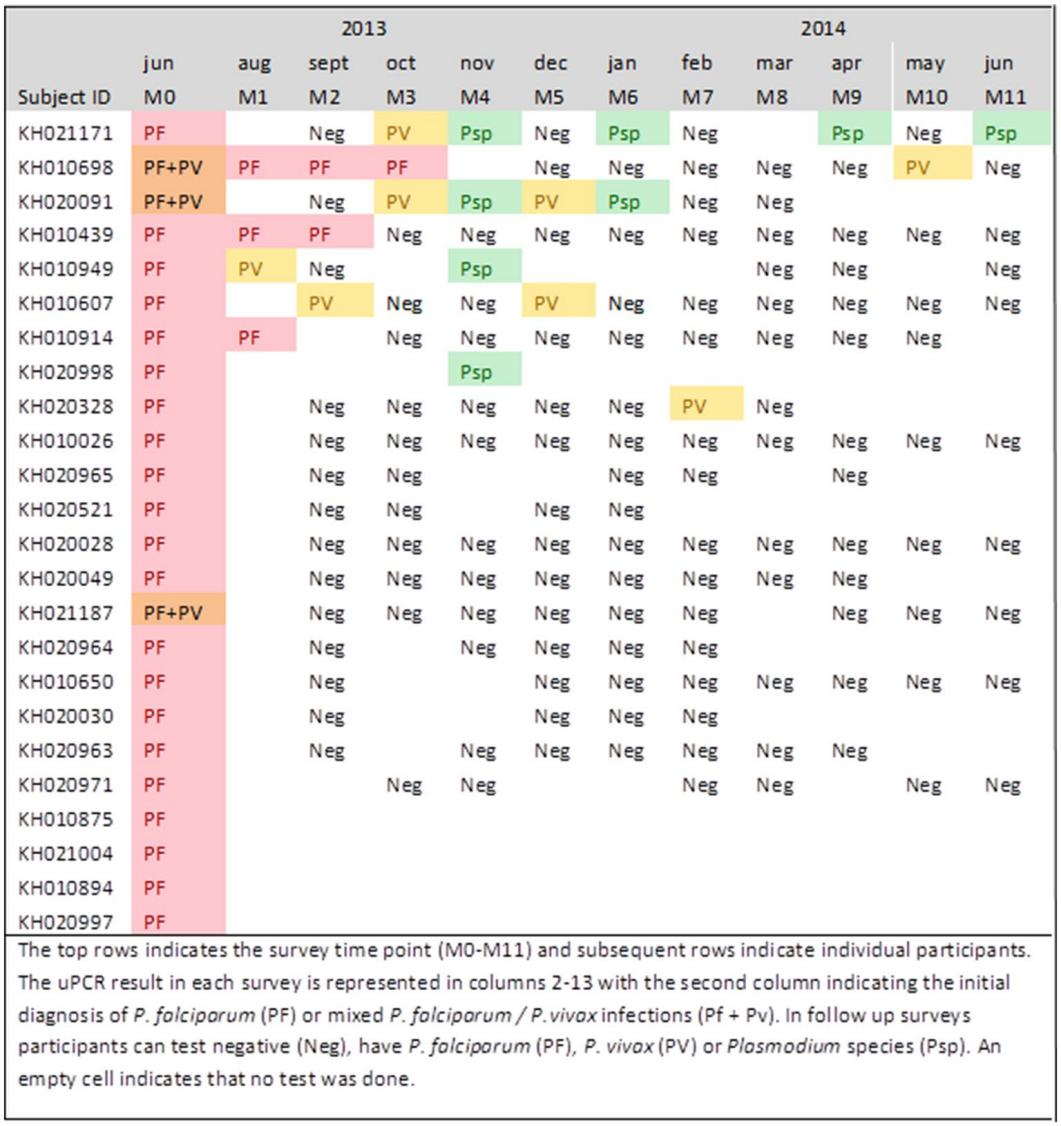
The persistence and transitions of *Plasmodium falciparum* infections in an adult cohort (ordered by number of episodes)

**Fig. 4:**
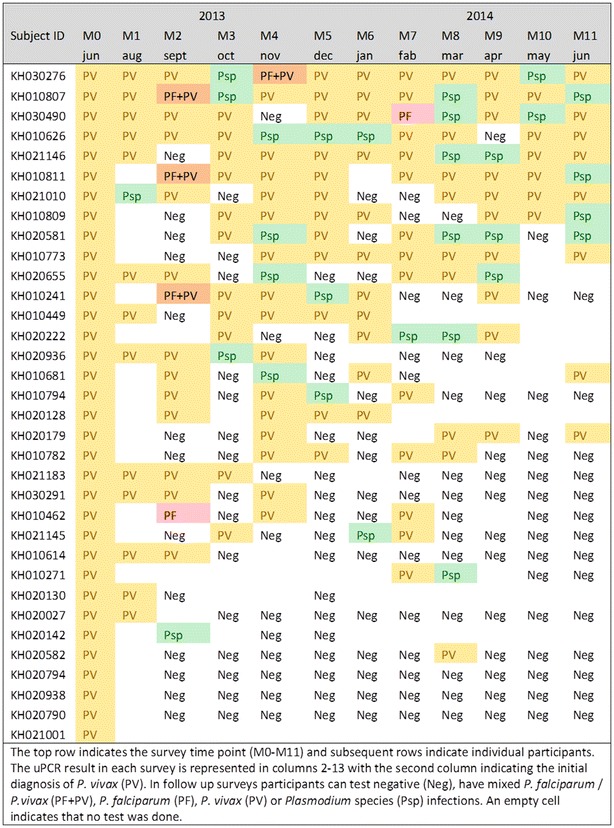
The persistence and transitions of *Plasmodium vivax* infections in an adult cohort (ordered by number of episodes)

136 villagers age 16 or older were found to be infected with *P. falciparum*, *P. vivax,* or *P. species* by uPCR in June 2013 (M0); 24 were infected with *P. falciparum*, 34 with *P. vivax* and in the rest the *Plasmodium* species could not be identified (n = 78). These 136 villagers were followed monthly for the 12 months surveillance period from June 2013 (M0) to June 2014 (M11). Of the 24 cohort members with mono or mixed *P. falciparum* infections at M0, three remained parasitaemic over 2–4 months, five were found have *P. vivax* parasitaemia during the follow-up period and four had *Plasmodium* infections in which the species could not be determined. The persistence of *P. falciparum* infections is illustrated in Fig. 3. Figure 4 shows the 34 cohort members with *P. vivax* mono-infections at M0; 12/34 (35 %) who were parasitaemic with *P. vivax* or *P. species* in more than half of the following surveys. The *Plasmodium* species of 78 participants found to be parasitaemic on M0 could not be determined (Additional file 1: Figure S1). Three of those 78 participants with *P. species* infections at M0 were parasitaemic with either *P. vivax* or *P. species* in at least half of the following surveys.

The risk of being positive for malaria by uPCR was investigated in the adult cohort (n = 136). The odds of carrying subpatent parasites decreased over time (AOR 0.82, 95 % CI 0.76–0.87). Being male and a self-reported history of malaria were the only two significant risk factors associated with a positive uPCR result (Table 3). Overall, a subgroup of 37 of the 136 cohort members was parasitaemic during four or more monthly surveys. Compared to other cohort members the carriers (Table 4) were significantly more likely to be male (26/37 vs. 45/99, p = 0.010) and were slightly older (median age 35 years) compared to 33 years in other cohort members (p = 0.373). All carriers were farmers by occupation (compared with 92/99 among non-carriers, p = 0.189).

**Table 3:** Comparison of variables associated with qPCR positivity in cohort members (n = 136)

	N	OR_crude	95 % CI	OR_adj^a^	95 % CI
Village					
KL	60	Ref.			
OK	17	0.99	0.42–2.35		
PDB	59	0.90	0.50–1.63		
Male	136	2.07	1.21–3.56	2.38	1.12–5.05
Age (median years; range)	136	1.00	0.98–1.02		
Farmer	136	2.72	0.76–9.70		
Bednet use (n = 124)	136	0.53	0.13–2.08		
History of fever	136	1.63	0.60–4.42		
History of malaria	136	99.0	32.1–306	92.1	29.3–290
Travel	131	3.88	1.60–9.41	2.63	0.92–7.56
Village age^a^					
KL	60	Ref.			
OK	17	0.98	0.91–1.05		
PDB	59	0.98	0.94–1.03		
Sex travel^a^	131	2.27	0.66–7.85		
Farmer age^a^	136	1.01	0.92–1.12		

^a^ Includes only variables that were significant in univariate analysis

**Table 4:** Comparison of cohort members who were prolonged parasite carriers and non-carriers

	Non-carriers	%	Carriers	%	Total	p value
N	99	73 %	37	27 %	136	
Village						
KL	43	71.7	17	28.3	60	
OK	11	64.7	6	35.3	17	
PDB	45	76.3	14	23.7	59	0.62*
Male	45	45.5 %	26	70.3 %	71	0.010
Age (median years; range)	33	16–72	35	16–65	34	0.37
Farmer	92	92.9 %	37	100 %	129	0.19

* 2 df

### Sub patent parasite densities

**Fig. 5:**
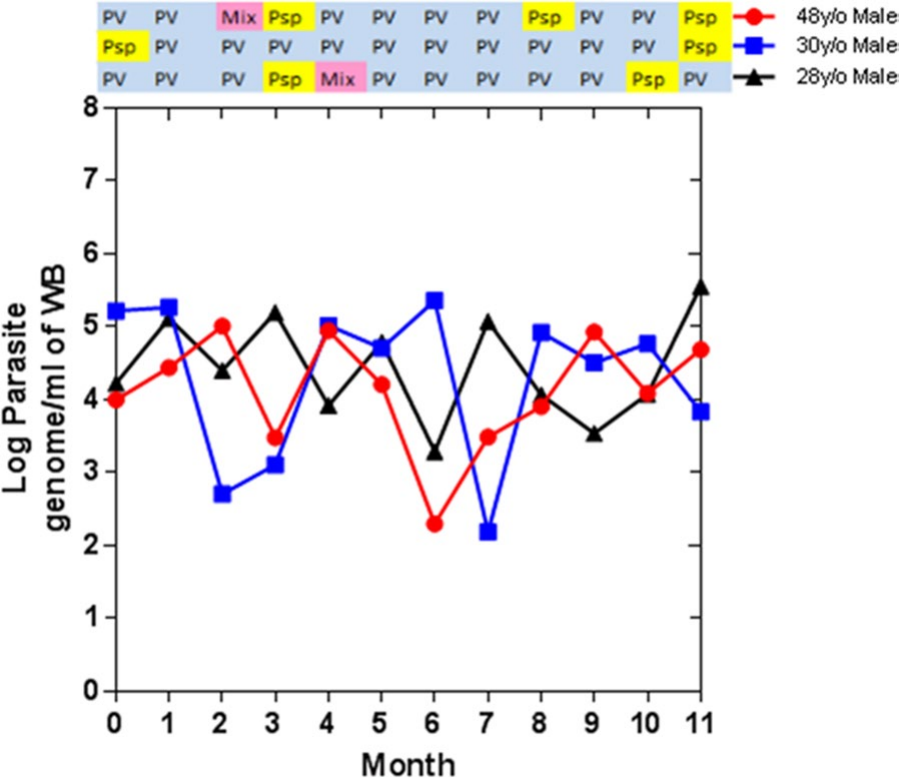
Log parasite densities (log parasites/mL) in three participants with persistent *Plasmodium vivax* infections

The individual parasite densities of the 37 carriers over the 12 months study period are shown in Additional file 1: Figure S2. Over the 12 months study periods parasitaemias were cleared between zero and four times (mean 3.3, standard deviation 1.3) and recurred between 0 and 4 times (mean 3.1, SD 1.2). Parasitaemia episodes lasted between 0 and 12 months (mean 2.8, SD 2.1). The periods of clearance in this cohort lasted between 1 and 8 months (mean 2.1, SD 1.8). In three carriers (KH10807, KH30276, KH21144; see Additional file 1: Figure S2), all male, parasites were detected in 12 sequential visits (Fig. 5). The three persistent carriers controlled the parasite densities within 2–4 logs (log parasites/mL). Two of the three carriers (KH10807, KH30276) had a sporadic co-infection (PV+PF) during the study period. Frequently parasites became undetectable during the study period either to stay undetectable during the remainder of the study period or to reappear at a later stage.

## Discussion

The availability of a new diagnostic tool, highly sensitive PCR (uPCR) provides a new perspective on the epidemiology of malaria, allowing characterization of the subclinical and submicroscopic *Plasmodium* reservoir [17]. The limit of parasite detection in this study is approximately 50 times lower than with the extensively employed capillary blood filter paper based PCR methods [36]. This is sufficient to identify the majority of infected individuals [37]. Approximately four times more asymptomatic infected individuals were identified in these studies with this method than with conventional microscopy or rapid diagnostic tests [36].

The study was conducted in an area of low seasonal malaria transmission at a time of strengthening malaria control and environmental change. The prevalence of *P. falciparum* infections was already low and declined further while the prevalence of *P. vivax* remained relatively stable. During the 1 year of follow up no obvious seasonality was detected. This disparity between the incidence of malaria and the prevalence of asymptomatic parasitaemia is also seen in areas of much higher transmission, such as the sub-Sahel, where there is also marked seasonality in the incidence of clinical malaria yet much less variation in asymptomatic carriage [38, 39]. This illustrates the importance of asymptomatic malaria as the transmission reservoir infecting increasing numbers of anopheline vectors, as vectorial capacity rises abruptly at the beginning of the rainy season.

Ongoing molecular studies strongly suggest that the observed low densities of parasites are alive as they are expressing RNA. There is debate whether low-density parasitaemias are transmissible [4]. Several studies from sub-Saharan Africa, Asia, South America, and Pacific Islands have demonstrated that submicroscopic falciparum as well as vivax infections can be transmitted to mosquitoes [40–47]. These discussions usually revolve around the transmission potential at the time of sampling, but would be more epidemiologically relevant if they considered the transmission potential of an individual over time. An individual may have a low-density non-transmissible infection on 1 day, but later densities may rise and infect biting anopheline mosquitoes. A recent study conducted in Papua New Guinea detected *P. falciparum* in 19 % and *P. vivax* in 13 % of 2083 samples using conventional PCR. Gametocytes could be detected in 60 % of *P. falciparum*-positive and 51 % of *P. vivax*-positive samples by detection of pfs25 and pvs25mRNA transcripts [48]. Gametocytaemia is probably present in all chronic infections but densities fluctuate out of phase with asexual parasitaemia waves.

A cohort of adults with *Plasmodium* parasitaemia at the time of the first survey was followed over a 12 months period in monthly intervals. In this cohort *P. falciparum* infections were short lived; three participants remained infected for 2–4 months. By contrast a third of the participants with *P. vivax* infections remained parasitaemic for more than half of the following surveys. Three different patterns of persistence were observed. First, the infection could be cleared within the first months of the study period and did not reoccur. Second, no parasitaemias were detected during 1 or 2 months, only to reappear during subsequent months. Third, in a minority of participants persistent parasitaemias could be detected during each survey. Changes in genotype profiles over time within individual participants are currently under investigation.

In participants who control their infections relatively well, as illustrated by three participants who remained parasitaemic and asymptomatic during 12 surveys, the parasite densities remain low and the oscillation amplitude was less than one hundred fold. Oscillating parasite densities have been described previously in vivax as well as falciparum malaria therapy for patients with neurosyphilis [14, 49] and a more recent study conducted in an area of PNG with very high *P. falciparum, P. vivax* and *P. malariae* prevalence [12]. In PNG 70 asymptomatic villagers were sampled every third day for a period of 61 days. Infections often lasted for several weeks in young children but generally for only a few days in adults. The periodicity of infections observed in PNG was attributed to sequestration of synchronously replicating *P. falciparum* parasites as reported elsewhere [50]. Periodic oscillation of *P. falciparum* densities has also been reported in asymptomatic children in rural Tanzania sampled daily for 14 days [51]. It seems more likely that parasite multiplication varies over time, controlled by mechanisms such as antibody responses to variant surface antigens [52, 53].

The decline in *P. falciparum* prevalence observed in this study could be short-lived. Half of the *P. falciparum* strains had a mutation (C580Y) in the gene coding for the K-13 propeller which is associated with reduced susceptibility to artemisinin derivatives. Artemisinins used in combination with a partner drug are the global first line treatment for uncomplicated malaria. In the presence of artemisinin resistance *P. falciparum* strains with reduced susceptibility of the partner drug emerge and spread rapidly [22, 23, 54]. The complete replacement of wild type with C580Y mutant strains in one village suggests a need for urgent action to interrupt the transmission of the remaining *P. falciparum* strains [55, 56].

The study also shows a shift in malaria burden from *P. falciparum* to *P. vivax* which is increasingly responsible for malaria related morbidity and mortality in the region. The radical cure of patients with persistent vivax infections has a high priority not only for the benefit of the patient but to minimize the transmission of this infection. Radical cure of vivax infections requires use of 8-aminoquinolines such as primaquine. Fear of haemolysis in G6PD deficient patients and the absence of a practical, low-cost rapid diagnostic test to diagnose G6PD deficiency have so far obstructed the wider use of this class of drugs.

## Conclusion

Malaria control in western Cambodia has been highly successful resulting in a more than a 100-fold reduction in *P. falciparum* cases and a more modest fivefold reduction in *P. vivax* cases in the study sites. The slower reduction in *P. vivax* than in *P. falciparum* transmission is likely due to the hypnozoite reservoir, which is resistant to current control measures. A new technology, uPCR, provides insights into the dynamics of asymptomatic, subpatent *Plasmodium* infections. The findings suggest that the elimination of submicroscopic reservoirs is an essential step in the eradication of malaria. The inclusion of hypnozoiticidal drugs such as primaquine in the first-line treatment of *P. vivax* malaria will be necessary to accelerate the elimination of vivax malaria.

## Additional file

10.1186/s12936-016-1224-7 The persistence and transitions of *Plasmodium* infections in which the species could not be identified.   **Figure S2.** The log parasite density of all 37 parasite carriers defined here as parasitaemic during 4 or more of the 12 monthly surveys (sorted by ID number).
