# Supplementary material for: Persistent Plasmodium falciparum and Plasmodium vivax infections in a western Cambodian population: implications for prevention, treatment and elimination strategies
Source: Malar J. 2016 Mar 24;15:181. doi: 10.1186/s12936-016-1224-7 (PMC4806483; doi:10.1186/s12936-016-1224-7)
Supplement: Supplementary file 1 — 10.1186/s12936-016-1224-7 The persistence and transitions of Plasmodium infections in which the species could not be identified. Figure S2. The log parasite density of all 37 parasite carriers defined here as parasitaemic during 4 or more of the 12 monthly surveys (sorted by ID number). [file 12936_2016_1224_MOESM1_ESM.pdf]

Additional figures

Additional file: Figure S1 The persistence and transitions of *Plasmodium* infections in which the species could not be identified.

	2013						2014					
	jun	aug	sept	oct	nov	dec	jan	feb	mar	apr	may	jun
Subject ID	M0	M1	M2	M3	M4	M5	M6	M7	M8	M9	M10	M11
KH021144	Psp	PV	PV	PV	PV	PV	PV	PV	PV	PV	PV	Psp
KH010795	Psp	PV	PV	Neg	PV	PV	PV	Neg	Neg	PV	Psp	Psp
KH020727	Psp		Neg		Neg	Neg	PV	PV	PV	PV	PV	PV
KH030079	Psp	PV	PV	PV	Neg	Psp	Psp	Neg	Neg	Neg		Neg
KH030046	Psp		PV	Psp	PV	PV	PV			Neg		
KH030169	Psp	PV	PV		PV	Neg	PV			Psp		
KH010702	Psp		PV	Neg	Neg	Psp		PV	Neg	Neg		Psp
KH021028	Psp				Psp		PV	Neg	Psp		Neg	PV
KH021078	Psp	PV	Neg	PF	Neg	PV	Neg	Neg	Neg	Neg	Neg	Neg
KH021060	Psp	PV	PV	PV	Neg		Neg		Neg	Neg	Neg	Neg
KH020735	Psp	PV	Neg	Neg	Neg	Neg	PV	Neg	Neg	Neg	Neg	Neg
KH030413	Psp		Neg	PV	Neg	Neg	Neg	PF	Neg	Neg	Neg	Neg
KH010446	Psp		Neg	Psp	Neg	PV	Neg					Neg
KH010400	Psp		Neg	Neg	Neg	Psp	Neg	Neg	Neg	Neg	Neg	Neg
KH030286	Psp		PV	Neg	Neg	Neg	Neg	Neg	Neg	Neg	Neg	Neg
KH010416	Psp	PV	Neg	Neg	Neg	Neg	Neg	Neg	Neg	Neg	Neg	Neg
KH030011	Psp	PF	Neg				Neg	Neg	Neg	Neg	Neg	Neg
KH010648	Psp		PV	Neg	Neg							
KH010071	Psp	PF		Neg								
KH010077	Psp		Neg	Neg	Neg	Neg	Neg	Neg	Neg	Neg	Neg	Neg
KH020340	Psp		Neg	Neg	Neg	Neg	Neg	Neg	Neg	Neg	Neg	Neg
KH010876	Psp		Neg	Neg	Neg	Neg	Neg	Neg	Neg	Neg	Neg	Neg
KH030330	Psp		Neg	Neg	Neg	Neg	Neg	Neg	Neg	Neg	Neg	Neg
KH021011	Psp		Neg	Neg	Neg	Neg	Neg	Neg	Neg	Neg	Neg	Neg
KH030473	Psp		Neg	Neg	Neg	Neg	Neg	Neg	Neg	Neg	Neg	Neg
KH030334	Psp		Neg	Neg	Neg	Neg	Neg	Neg	Neg	Neg	Neg	Neg
KH030323	Psp		Neg	Neg	Neg	Neg	Neg	Neg	Neg	Neg	Neg	Neg
KH010623	Psp		Neg	Neg	Neg	Neg	Neg	Neg	Neg	Neg	Neg	Neg
KH030348	Psp		Neg	Neg	Neg	Neg	Neg	Neg	Neg	Neg	Neg	Neg
KH010584	Psp		Neg	Neg	Neg	Neg	Neg	Neg	Neg	Neg	Neg	Neg
KH010011	Psp		Neg	Neg	Neg	Neg	Neg	Neg		Neg	Neg	Neg
KH010196	Psp		Neg	Neg	Neg	Neg	Neg	Neg	Neg	Neg	Neg	Neg
KH020040	Psp		Neg	Neg	Neg	Neg	Neg	Neg	Neg	Neg	Neg	Neg
KH010029	Psp		Neg	Neg	Neg	Neg	Neg	Neg	Neg	Neg	Neg	Neg
KH030282	Psp		Neg	Neg	Neg	Neg	Neg	Neg	Neg	Neg	Neg	Neg
KH020231	Psp		Neg	Neg	Neg	Neg	Neg	Neg	Neg	Neg	Neg	Neg
KH010197	Psp		Neg	Neg	Neg	Neg	Neg	Neg	Neg	Neg	Neg	Neg
KH020239	Psp		Neg	Neg	Neg	Neg	Neg	Neg	Neg	Neg	Neg	Neg
KH030407	Psp		Neg	Neg	Neg	Neg	Neg	Neg	Neg	Neg	Neg	Neg
KH020068	Psp		Neg	Neg	Neg	Neg	Neg	Neg	Neg	Neg	Neg	Neg
KH020937	Psp		Neg	Neg	Neg	Neg	Neg	Neg	Neg	Neg	Neg	Neg

KH010103	Psp		Neg	Neg	Neg	Neg	Neg	Neg	Neg	Neg	Neg	Neg
KH010270	Psp		Neg	Neg	Neg	Neg		Neg	Neg		Neg	Neg
KH010591	Psp		Neg	Neg	Neg	Neg	Neg	Neg	Neg	Neg		Neg
KH010056	Psp		Neg	Neg	Neg	Neg	Neg	Neg	Neg		Neg	Neg
KH030002	Psp		Neg	Neg	Neg	Neg	Neg		Neg	Neg	Neg	Neg
KH010945	Psp		Neg	Neg	Neg	Neg	Neg	Neg	Neg	Neg		Neg
KH021012	Psp		Neg	Neg	Neg	Neg	Neg	Neg	Neg		Neg	Neg
KH010787	Psp		Neg	Neg		Neg	Neg	Neg	Neg	Neg		Neg
KH020466	Psp		Neg	Neg	Neg	Neg	Neg	Neg				Neg
KH020422	Psp		Neg	Neg	Neg	Neg			Neg		Neg	Neg
KH010718	Psp		Neg	Neg	Neg	Neg	Neg	Neg			Neg	Neg
KH010596	Psp		Neg	Neg	Neg	Neg	Neg	Neg	Neg	Neg		
KH010220	Psp		Neg	Neg	Neg	Neg				Neg	Neg	Neg
KH010717	Psp		Neg	Neg	Neg	Neg	Neg	Neg				Neg
KH020660	Psp				Neg	Neg	Neg	Neg		Neg	Neg	Neg
KH010218	Psp		Neg				Neg	Neg	Neg	Neg	Neg	Neg
KH020331	Psp		Neg	Neg	Neg	Neg	Neg				Neg	Neg
KH010913	Psp			Neg	Neg	Neg	Neg	Neg	Neg	Neg	Neg	
KH020970	Psp		Neg	Neg	Neg		Neg	Neg	Neg			
KH010049	Psp		Neg	Neg	Neg					Neg	Neg	Neg
KH010070	Psp		Neg	Neg	Neg					Neg	Neg	Neg
KH021048	Psp		Neg	Neg	Neg	Neg	Neg	Neg				
KH010912	Psp				Neg	Neg		Neg	Neg		Neg	Neg
KH020532	Psp		Neg	Neg	Neg				Neg			Neg
KH010260	Psp		Neg	Neg	Neg	Neg	Neg					
KH010073	Psp			Neg	Neg	Neg				Neg	Neg	
KH010873	Psp			Neg	Neg	Neg						Neg
KH010957	Psp		Neg		Neg	Neg				Neg		
KH020981	Psp				Neg			Neg			Neg	Neg
KH021109	Psp						Neg					
KH021046	Psp			Neg								
KH020786	Psp		Neg									
KH021035	Psp				Neg							
KH021093	Psp											
KH010895	Psp											
KH010082	Psp											
KH010052	Psp											

The top row indicates the survey time point (M0-M11) and subsequent rows indicate individual participants. The uPCR result in each survey is represented in columns 2-13 with the second column indicating the initial diagnosis of *Plasmodium* species (Psp). In follow up surveys participants can test negative (Neg), *P. falciparum* (PF) or *P. vivax* (PV). An empty cell indicates that no test was done.

Additional file 1: Figure S2 The log parasite density of all 37 parasite carriers defined here as parasitaemic during 4 or more of the 12 monthly surveys (sorted by ID number). Each cell represents an individual participant. The three highlighted participants are also shown in Figure 5.

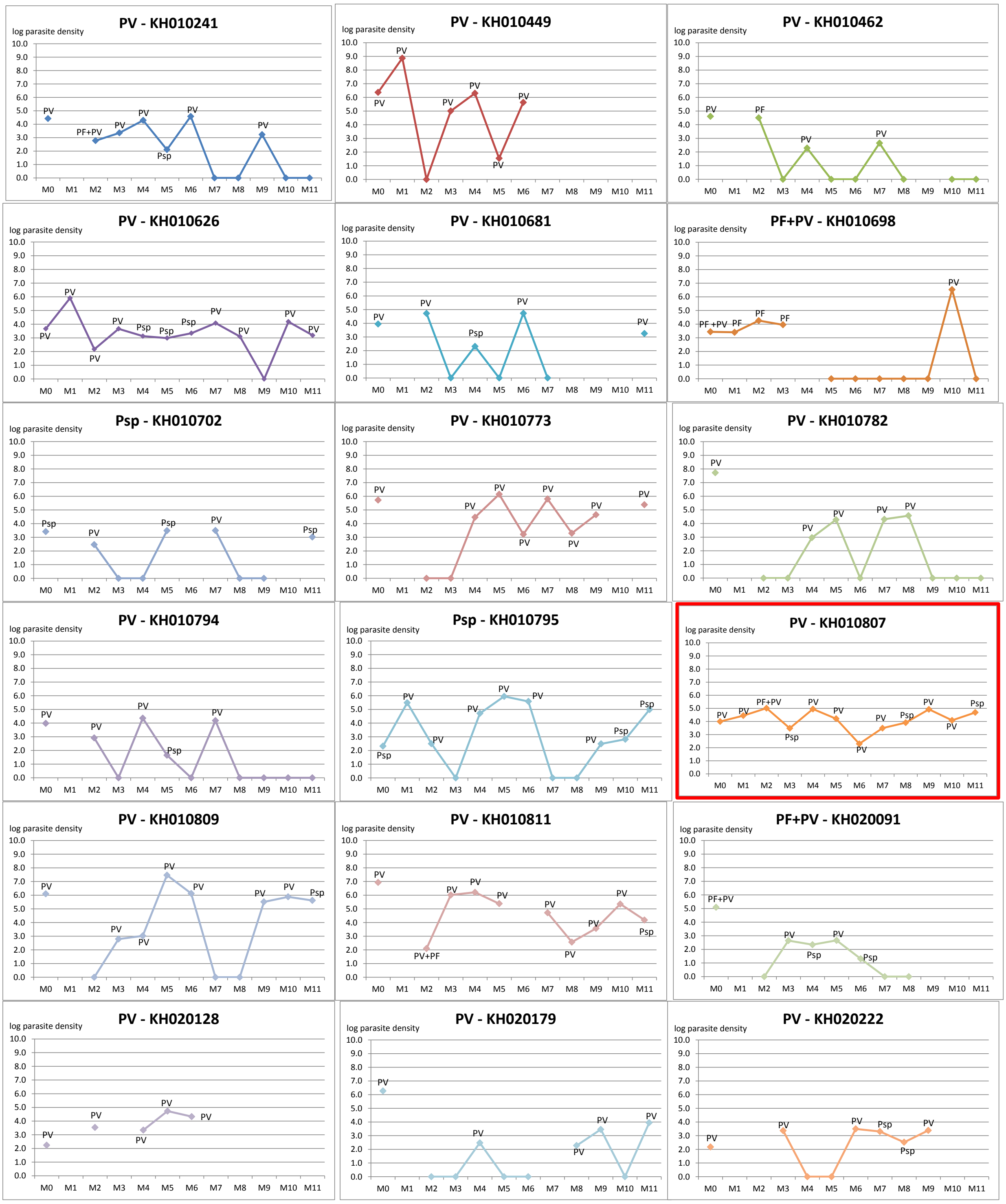

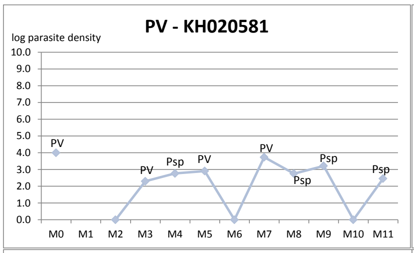
PV - KH020581
log parasite density
0.0 1.0 2.0 3.0 4.0 5.0 6.0 7.0 8.0 9.0 10.0
M0 M1 M2 M3 M4 M5 M6 M7 M8 M9 M10 M11
PV PV Psp PV PV Psp Psp Psp

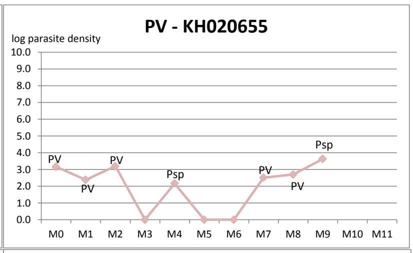
PV - KH020655
log parasite density
0.0 1.0 2.0 3.0 4.0 5.0 6.0 7.0 8.0 9.0 10.0
M0 M1 M2 M3 M4 M5 M6 M7 M8 M9 M10 M11
PV PV PV Psp PV PV Psp

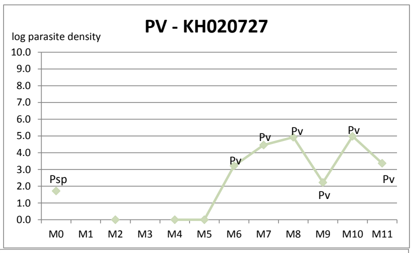
PV - KH020727
log parasite density
0.0 1.0 2.0 3.0 4.0 5.0 6.0 7.0 8.0 9.0 10.0
M0 M1 M2 M3 M4 M5 M6 M7 M8 M9 M10 M11
Psp Pv Pv Pv Pv Pv Pv

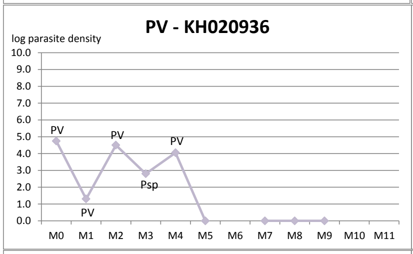
PV - KH020936
log parasite density
0.0 1.0 2.0 3.0 4.0 5.0 6.0 7.0 8.0 9.0 10.0
M0 M1 M2 M3 M4 M5 M6 M7 M8 M9 M10 M11
PV PV PV Psp PV

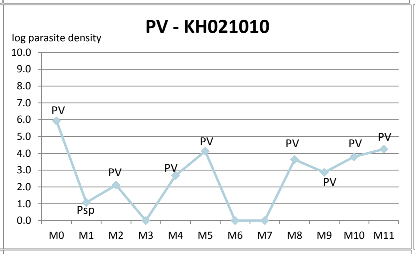
PV - KH021010
log parasite density
0.0 1.0 2.0 3.0 4.0 5.0 6.0 7.0 8.0 9.0 10.0
M0 M1 M2 M3 M4 M5 M6 M7 M8 M9 M10 M11
PV Psp PV PV PV PV PV PV PV

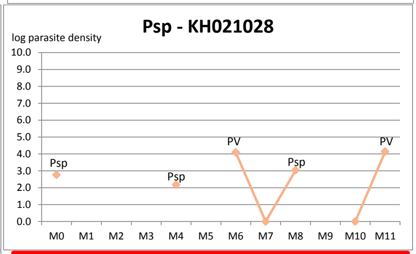
Psp - KH021028
log parasite density
0.0 1.0 2.0 3.0 4.0 5.0 6.0 7.0 8.0 9.0 10.0
M0 M1 M2 M3 M4 M5 M6 M7 M8 M9 M10 M11
Psp Psp PV Psp PV

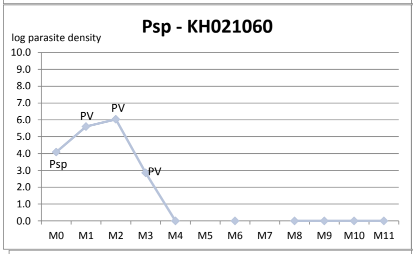
Psp - KH021060
log parasite density
0.0 1.0 2.0 3.0 4.0 5.0 6.0 7.0 8.0 9.0 10.0
M0 M1 M2 M3 M4 M5 M6 M7 M8 M9 M10 M11
Psp PV PV PV

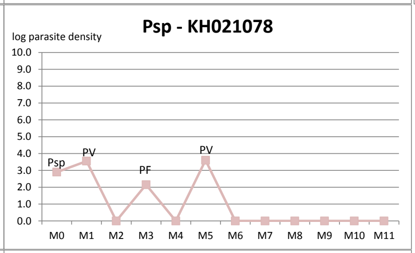
Psp - KH021078
log parasite density
0.0 1.0 2.0 3.0 4.0 5.0 6.0 7.0 8.0 9.0 10.0
M0 M1 M2 M3 M4 M5 M6 M7 M8 M9 M10 M11
Psp PV PF PV

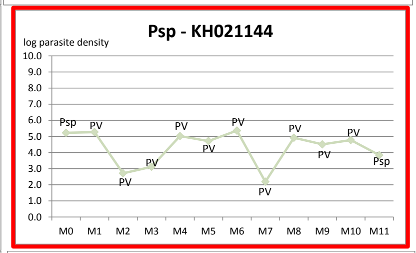
Psp - KH021144
log parasite density
0.0 1.0 2.0 3.0 4.0 5.0 6.0 7.0 8.0 9.0 10.0
M0 M1 M2 M3 M4 M5 M6 M7 M8 M9 M10 M11
Psp PV PV PV PV PV PV PV PV PV PV Psp

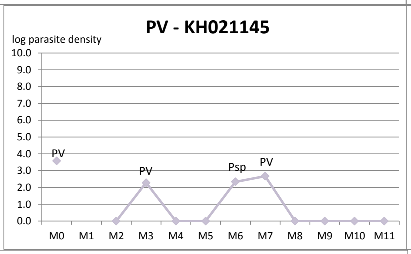
PV - KH021145
log parasite density
0.0 1.0 2.0 3.0 4.0 5.0 6.0 7.0 8.0 9.0 10.0
M0 M1 M2 M3 M4 M5 M6 M7 M8 M9 M10 M11
PV PV Psp PV

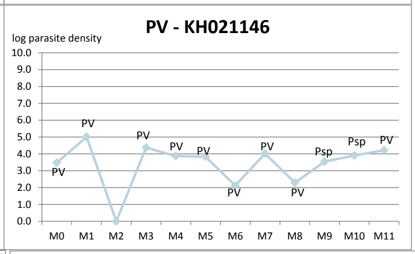
PV - KH021146
log parasite density
0.0 1.0 2.0 3.0 4.0 5.0 6.0 7.0 8.0 9.0 10.0
M0 M1 M2 M3 M4 M5 M6 M7 M8 M9 M10 M11
PV PV PV PV PV PV PV PV Psp Psp PV

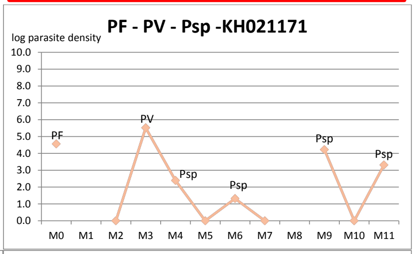
PF - PV - Psp -KH021171
log parasite density
0.0 1.0 2.0 3.0 4.0 5.0 6.0 7.0 8.0 9.0 10.0
M0 M1 M2 M3 M4 M5 M6 M7 M8 M9 M10 M11
PF PV Psp Psp Psp Psp

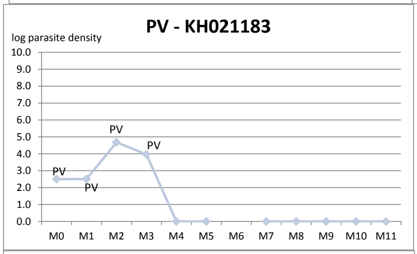
PV - KH021183
log parasite density
0.0 1.0 2.0 3.0 4.0 5.0 6.0 7.0 8.0 9.0 10.0
M0 M1 M2 M3 M4 M5 M6 M7 M8 M9 M10 M11
PV PV PV PV

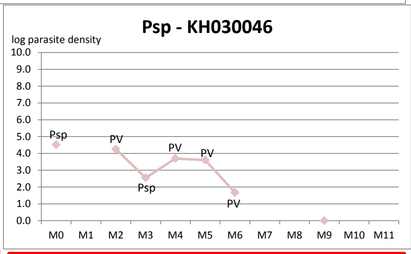
Psp - KH030046
log parasite density
0.0 1.0 2.0 3.0 4.0 5.0 6.0 7.0 8.0 9.0 10.0
M0 M1 M2 M3 M4 M5 M6 M7 M8 M9 M10 M11
Psp PV Psp PV PV PV

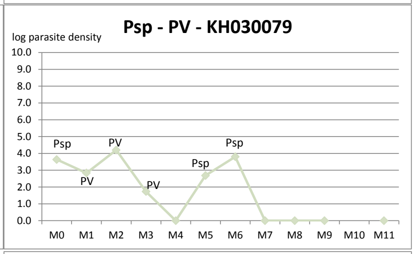
Psp - PV - KH030079
log parasite density
0.0 1.0 2.0 3.0 4.0 5.0 6.0 7.0 8.0 9.0 10.0
M0 M1 M2 M3 M4 M5 M6 M7 M8 M9 M10 M11
Psp PV PV PV Psp Psp

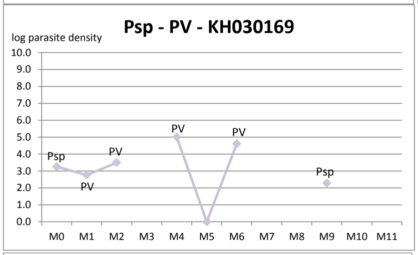
Psp - PV - KH030169
log parasite density
0.0 1.0 2.0 3.0 4.0 5.0 6.0 7.0 8.0 9.0 10.0
M0 M1 M2 M3 M4 M5 M6 M7 M8 M9 M10 M11
Psp PV PV PV PV Psp

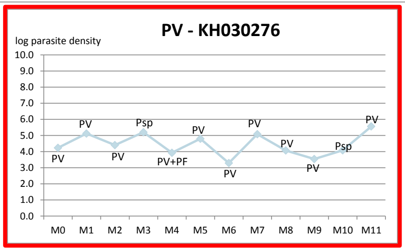
PV - KH030276
log parasite density
0.0 1.0 2.0 3.0 4.0 5.0 6.0 7.0 8.0 9.0 10.0
M0 M1 M2 M3 M4 M5 M6 M7 M8 M9 M10 M11
PV PV PV Psp PV+PF PV PV PV PV PV Psp PV

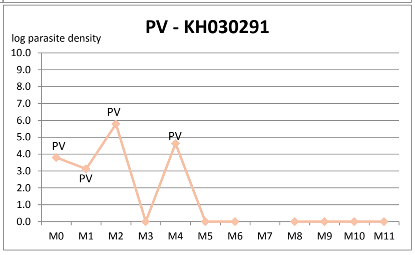
PV - KH030291
log parasite density
0.0 1.0 2.0 3.0 4.0 5.0 6.0 7.0 8.0 9.0 10.0
M0 M1 M2 M3 M4 M5 M6 M7 M8 M9 M10 M11
PV PV PV PV

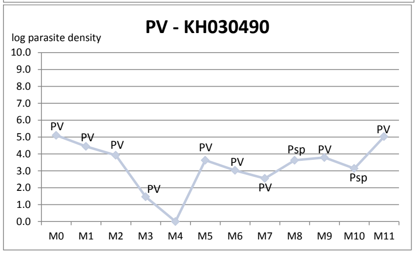
PV - KH030490
log parasite density
0.0 1.0 2.0 3.0 4.0 5.0 6.0 7.0 8.0 9.0 10.0
M0 M1 M2 M3 M4 M5 M6 M7 M8 M9 M10 M11
PV PV PV PV PV PV PV Psp PV Psp PV
